# Associations between Health Interest Scale Dimensions and Obesity Risk: A Cross-sectional Study among Japanese Employees

**DOI:** 10.31662/jmaj.2024-0388

**Published:** 2025-03-28

**Authors:** Yumiko Iwase, Rikuya Hosokawa

**Affiliations:** 1 Department of Human Health Sciences, Graduate School of Medicine, Kyoto University, Kyoto, Japan

**Keywords:** attitude to health, health behaviour, consciousness, obesity, occupational health

## Abstract

**Introduction:**

Obesity affects over 2.5 billion adults globally in 2022, posing a significant public health challenge. In Japan, obesity, defined as a body mass index ≥25 kg/m^2^, impacted 33.0% of men and 22.3% of women in 2020. Despite this, over 25% of Japanese adults report no intention to improve health habits.

The Health Interest Scale (HIS) assesses health-related attitudes across three dimensions: health consciousness, health motivation, and health value. Although overall HIS scores are associated with health outcomes, the specific roles of these dimensions in obesity risk are unclear. This study examines the associations between each HIS dimension and obesity risk among Japanese corporate employees, supporting targeted interventions for sedentary, working-age populations.

**Methods:**

This cross-sectional study analyzed data from 2,260 employees of information technology-related companies in Japan, collected via health checkups and self-administered surveys in 2023. HIS scores (range: 0-36) were used as continuous variables. Logistic regression assessed associations between HIS subscale scores and obesity status, adjusting for demographic, occupational, and lifestyle factors and obesity-related diseases.

**Results:**

Higher scores on each HIS dimension were associated with lower obesity odds. Adjusted odds ratios per one-point increase were: health consciousness, 0.84 (95% confidence interval: 0.81-0.88); health value, 0.85 (0.81-0.89); and health motivation, 0.91 (0.87-0.95). Male sex, short sleep (<7 hours), and sedentary occupations were associated with increased obesity odds (all p < 0.001). The associations remained significant after adjustment for obesity-related diseases. Additionally, stronger associations were observed among participants with obesity-related diseases in univariate analysis.

**Conclusions:**

This study identified significant associations between the three HIS dimensions and obesity risk, which remained robust after adjusting for obesity-related diseases. Stronger associations were observed across all HIS dimensions in participants with obesity-related diseases. These findings underscore the importance of tailored interventions targeting HIS dimensions, particularly health consciousness and health value, to reduce obesity risk in sedentary, working-age populations.

## Introduction

As of 2022, more than 2.5 billion adults worldwide, representing 43% of those aged ≥18 years, are overweight, and 890 million adults (16%) have obesity. The global obesity rate has approximately doubled since 1990 ^(1)^, and the World Obesity Federation predicts that approximately one in four people worldwide will become obese by 2035 ^(2)^. Furthermore, from 1990 to 2022, the proportion of countries where obesity prevalence exceeded underweight prevalence was projected to reach 89% for women and 73% for men ^(3)^. Obesity is associated with non-communicable diseases, such as type 2 diabetes, cardiovascular diseases, hypertension, stroke, cancer, and mental health issues, making the establishment of prevention and intervention strategies an urgent priority ^(1)^.

Globally, obesity is defined as a body mass index (BMI) of ≥30 kg/m^2^, whereas in Japan, obesity is defined as a BMI of ≥25 kg/m^2^ ^(4)^. Based on this standard, Japan’s obesity rate in 2020 was 33.0% among adult men and 22.3% among adult women, making obesity a major public health issue ^(5)^. The 2020 National Health and Nutrition Survey in Japan revealed that many adults were indifferent to improving their lifestyles. Specifically, 24.6% of men and 25.0% of women reported no intention to improve their dietary habits, whereas 23.9% of men and 26.3% of women expressed no intention to increase their physical activity levels ^(5)^. Moreover, individuals indifferent to health often mistakenly believe their lifestyle behaviors are adequately healthy ^(6)^. Therefore, effective interventions targeting indifferent populations are urgently required. Japan’s “Health Japan 21 (Third Edition),” a national health promotion policy framework aimed at extending healthy life expectancy and reducing health disparities, also identified addressing health indifference as a critical issue ^(7)^.

Intervention approaches focusing on high-risk individuals and reaching those at lower risk through population-based strategies have been widely recommended ^(8), (9)^. However, it has been pointed out that vulnerable populations, including those with low health interest, often respond poorly to interventions aimed at improving lifestyle behaviors ^(10), (11)^. Wakabayashi et al. ^(12)^ (2023) reported that individuals with low health interests are more vulnerable to health problems such as problem drinking, regardless of socioeconomic status. This underscores the need for tailored strategies to improve health outcomes in this population. Previous studies have examined conceptual frameworks associated with health behaviors ^(13)^. The measurement of health consciousness has also been developed and validated as a tool to assess health-related attitudes and behaviors ^(14)^. The Health Interest Scale (HIS) was subsequently developed to comprehensively assess health interests and identify individuals with low health engagement. This scale has shown high reliability and validity ^(15)^. HIS is a multidimensional tool comprising three subscales―health consciousness, health motivation, and health value―each representing unique dimensions of health-related attitudes and behaviors. The developers of the HIS emphasize the importance of conducting further research in diverse populations, such as workplaces and communities, to strengthen its external validity by comparing findings across various contexts. They also highlight the need for larger-scale studies to examine the associations between health interests, health behaviors, socioeconomic status, and work-related factors.

Previous studies have primarily examined overall HIS scores in association with health outcomes, leaving the specific contributions of each subscale to issues like obesity unclear. Examining the individual subscales enables a better understanding of how different aspects of health interest are associated with obesity risk, potentially offering insights for tailored intervention strategies.

This study focuses on employees in information technology-related industries who predominantly engage in sedentary work and represent the working-age population. This group often faces limited opportunities to prioritize health as a result of the demands of managerial or high-responsibility roles. Accordingly, this study aimed to assess the associations between each HIS subscale and obesity risk among Japanese corporate employees. Recognizing that individuals without obesity-related diseases may be less inclined to engage in lifestyle improvements, this study included occupational and lifestyle factors, along with obesity-related diseases, as covariates.

By analyzing these subscales individually, the study seeks to identify which dimensions of health interest are most relevant to obesity prevention and to provide insights for developing targeted, effective interventions.

## Materials and Methods

### Study design and participants

This cross-sectional study was conducted among employees of a large information technology-related company located in Japan’s metropolitan area. Participants were those who responded to a self-administered questionnaire survey administered during their regular health check-up in the fiscal year 2023.

### Sample size

The sample size for this study was calculated based on an expected response rate of 60%, derived from a 2020 web survey conducted by the company on the teleworking environment. The sample size was estimated using the formula for simple random sampling.


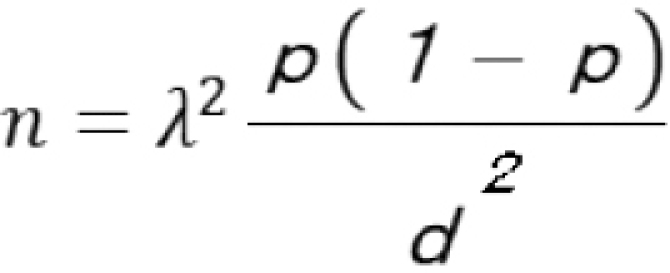


where *n* is the required sample size, *p* is the estimated response rate (0.6), *d* is the margin of error (0.05), and λ is the z-value corresponding to the 95% confidence level (1.96).

Based on this calculation, the minimum required sample size was determined to be 368 participants in this study. Considering that women comprised 18.7% of the workforce in the study setting in 2022, the total target sample size was increased to approximately 2,053 participants to enable meaningful subgroup analyses. This adjustment also accounted for potential non-responses and ensured adequate representation of both sexes within the sample.

### Outcome variables for obesity

Obesity was defined as the outcome variable to address the prevention of severe obesity-related diseases. The classification was used on the internationally recognized measure of BMI, calculated as [weight (kg)] / [height (m)^2^]. Individuals with a BMI ≥25 kg/m^2^ were classified as obese.

### Explanatory variables

The HIS is a validated 12-item scale that assesses health interest across three subscales: health consciousness, motivation, and value. Each item is rated on a 4-point Likert scale (0-3), with reverse scoring for items 9-12. Total scores range from 0 to 36, with higher scores reflecting greater health interest. In this study, the scores for each subscale were used as independent variables and analyzed as continuous variables to examine their associations with obesity risk.

Supplementary Table S-1 provides detailed descriptions of the HIS items, including reliability analysis.

### Instrument reliability and validity

The HIS was validated by its developers, Ozawa et al. ^(15)^, demonstrating high internal consistency with a Cronbach’s alpha of 0.85 for the overall scale. Confirmatory factor analysis demonstrated good construct validity, with indices including a goodness-of-fit index of 0.932, an adjusted goodness-of-fit index of 0.896, a normed fit index of 0.936, and a root mean square error of approximation of 0.079. Convergent validity was supported by significant correlations between HIS scores and both Health Locus of Control (r = 0.55, p < 0.001) and general concern for personal health (r = 0.64, p < 0.001).

In this study, we reassessed the reliability of the HIS by calculating the mean, standard deviation (SD), item-total correlations, and Cronbach’s alpha for each subscale. The Cronbach’s alpha values for the subscales “health consciousness,” “health motivation,” and “health value” were 0.85, 0.78, and 0.69, respectively. Although the value for “health value” falls slightly below the commonly recommended threshold of 0.7, previous research suggests that such values can still be acceptable depending on the context ^(16)^. The overall scale demonstrated a Cronbach’s alpha of 0.86, confirming its high reliability.

### Covariates

The covariates included demographic characteristics, lifestyle factors, and obesity-related diseases, as follows:

Demographic data

Demographic characteristics were assessed using health check-up questionnaires and included sex, age, occupation, job position, presence of co-resident family members, and marital status. Occupations were categorized into two groups based on job activity: “more sedentary occupations” (clerical workers, system engineers, or customer engineers) and “less sedentary occupations” (sales, R and D positions, and managerial staff) ^(17)^. Job positions were classified into two categories: “general staff” (general staff, manager, and senior manager) and “management staff” (director, executive director, or above) ^(18)^. Responses on co-resident family members and marital status were dichotomized as “Yes” or “No,” as living alone is associated with a higher risk of obesity compared to living in multi-person households, and married individuals tend to have a higher obesity risk than unmarried ones ^(19)^.

Lifestyle behaviors

Given the established association between lifestyle behaviors and obesity, items related to lifestyle behaviors were extracted from questionnaires collected during health checkups and categorized based on previous research findings. Smoking status was categorized as “non-smoker,” “former smoker or smoker” ^(20)^. Breakfast consumption frequency was categorized as “daily,” “occasionally or none” ^(21)^. Snack consumption frequency was classified as “never,” “sometimes or everyday” ^(22)^. Physical activity was divided into “≥60 min of walking per day” or “< 60 min of walking per day” ^(23)^. Sleep duration was categorized as “≥7 h” or “<7 h” ^(24)^.

Obesity-related diseases

Hyperglycemia, hypertension, and dyslipidemia were categorized as “Yes” or “No,” based on self-reported diagnoses. Individuals who reported “Yes,” were classified as having the disease.

### Statistical analyses

#### Descriptive statistics

Descriptive statistics were used to summarize participant characteristics. Continuous variables are expressed as means ± SD or medians, depending on the distribution, while categorical variables are reported as frequencies and percentages.

Group differences in HIS subscale scores between obese and non-obese participants were evaluated using the Mann-Whitney U test due to the non-normal distribution of the data. Chi-square tests were employed to compare categorical variables, and independent sample t-tests were conducted for continuous variables with normal distributions. For comparisons across multiple groups, one-way analysis of variance followed by Tukey’s post-hoc test was performed.

#### Univariable linear regression

Univariable linear regression analyses were conducted to assess the crude associations between BMI (dependent variable) and each HIS subscale (independent variable). Regression coefficients (B), 95% confidence intervals, and p-values were reported.

#### Logistic regression analysis of HIS subscales and obesity

Multivariable logistic regression analyses were performed to evaluate the associations between HIS subscale scores (as continuous variables) and obesity (BMI ≥25 kg/m^2^). The models were adjusted for potential confounders, including demographic and lifestyle characteristics, as well as the presence of obesity-related diseases.

## Results

### Participant characteristics

**Figure 1.**
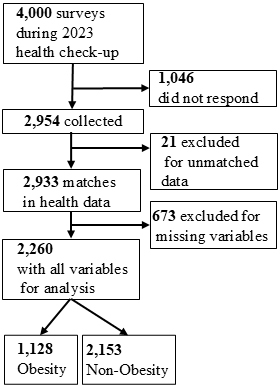
Flowchart of participant selection process. Flowchart depicting the steps for participant selection and categorization into obesity and non-obesity groups.

From 27 November 2023 to 17 January 2024, 4,000 questionnaires were distributed during the 2023 fiscal year health checkups and responses were received from 2,954 individuals (73.9%). After matching these responses to the health check-up data, 2,933 individuals were linked. After excluding 673 individuals with missing data, 2,260 participants were included in the analysis (Figure 1).

The participant characteristics are presented in Table 1. Of the 2,260 participants, 85.3% were male individuals, with a mean age of 50.68 ± 6.82 years. Consequently, 56.6% were in sedentary occupations. In terms of job positions, the majority (89.2%) were general staff, while management staff accounted for 10.8%. Regarding family composition, 73.3% had co-resident family members and 75.6% had a spouse. The BMI distribution, based on the World Health Organization (WHO) Obesity Classification, showed that 4.3% of participants were underweight, 61.9% had a normal BMI, and 27.0% were pre-obese. Obesity was observed in 6.7% of participants, including 5.4% in obese class I, 1.1% in class II, and 0.2% in class III. The mean BMI for all participants was 23.98 ± 3.82 kg/m^2^.

**Table 1.** Characteristics of the Study Participants.

(N＝2,260)						
Categorical variable	Categorisation criteria	Total N (%)	Obesity N (%)	Non-obesity N (%)	Effect size (Cramer’sV)	Chi-square test (*p*-value)
Sex, N (%)						
	Male	1928 (85.3%)	696 (91.3%)	1232 (82.2%)	0.12	**<0.001**
	Female	332 (14.7%)	66 (8.7%)	266 (17.8%)		
Occupational categories, N (%)						
	Less Sedentary occupations	980 (43.4%)	305 (40.0%)	675 (68.9%)	0.05	**<0.05**
	More Sedentary occupations	1280 (56.6%)	457 (60.0%)	823 (64.3%)		
Job/Position categories, N (%)						
	General staff	2015 (89.2%)	682 (89.5%)	1333 (89.0%)	0.01	0.78
	Management staff	245 (10.8%)	80 (10.5%)	165 (11.0%)		
Presence of co-resident family members, N (%)						
	Yes	1657 (73.3%)	526 (69.0%)	1131 (75.5%)	0.07	**<0.05**
	No	603 (26.7%)	236 (31.0%)	367 (24.5%)
Marital status, N (%)						
	Yes	1709 (75.6%)	553 (72.6%)	1156 (77.2%)	0.05	**<0.05**
	No	551 (24.4%)	209 (27.4%)	342 (22.8%)		
Age categories, N (%), years						
	≤39	115 (5.1%)	36 (4.7%)	79 (5.3%)	0.05	**<0.05**
	40-49	809 (36.0%)	247 (32.4%)	562 (37.5%)
	≥50	1336 (58.9%)	479 (62.9%)	857 (57.2%)
Obesity related disease, N (%)						
	Yes	473 (20.9%)	259 (34.0%)	214 (14.3%)	0.23	**<0.001**
	No	1787 (79.1%)	503 (66.0%)	1284 (85.7%)		

**WHO Obesity classification**	**BMI classification**	**N**	**(%)**			
	Underweight (<18.5)	98	(4.3%)			
	Normal (18.5-24.9)	1400	(61.9%)			
	Pre-obese (25.0-29.9)	611	(27.0%)			
	Obese Class I (30.0-34.9)	122	(5.4%)			
	Obese Class II (35.0-39.9)	24	(1.1%)			
	Obese Class III (≥40.0)	5	(0.2%)			

Continuous variable	Mean±SD					
Age, years	50.68±6.82					
BMI, kg/m^2^	23.98±3.82					
HIS Score	23.01±5.03					
Health Consciousness	7.53±2.12					
Health Motivation	7.59±1.99					
Health Value	7.90±2.02					

Occupational categories: “More sedentary occupations” include System engineer/Customer engineer (SE/CE) and Clerical workers. “Less sedentary occupations” include all other roles. Job position categories: “Management staff” includes director, Executive director, or higher positions. “General staff” includes all other positions. HIS score: The HIS is a validated 12-item scale that evaluates health interest across three subscales: health consciousness, health motivation, and health value. Each item is rated on a 4-point Likert scale (0-3), with reverse scoring applied to items 9-12. Total scores range from 0 to 36.

### Participant characteristics by obesity status

The characteristics of the participants according to their obesity status are presented in Table 1. Obesity was more prevalent in men than in women, as indicated by the chi-square test (p < 0.001), suggesting a small but significant association between sex and obesity status. Age was categorized into three groups (≤39, 40-49, and ≥50 years) for reanalysis. This categorization was based on evidence indicating a significant rise in the prevalence of metabolic syndrome among men, from 24.2% in their 30s to 42.7% in their 40s ^(5)^. This categorization revealed a significant association with obesity status (p* <* 0.05). Additionally, higher obesity rates were observed among those without co-resident family members (p < 0.05), and those without a spouse (p < 0.05). Supplementary Table S-2 provides detailed comparisons of HIS scores by participant characteristics.

### Comparison of HIS subscale scores between obese and non-obese groups

Table 2 presents the association between HIS subscale scores and obesity status. The Mann-Whitney U test indicated that mean scores for all three subscales were significantly higher in the non-obese group than in the obese group (p < 0.001). Specifically, the mean health consciousness score was 7.78 ± 2.17 in the non-obese group compared to 6.95 ± 2.22 in the obese group (p < 0.001). Similarly, health motivation scores were higher in the non-obese group (7.70 ± 1.99) than in the obese group (7.27 ± 1.97) (p < 0.001). Health value scores also followed this pattern, with the non-obese group scoring 8.15 ± 2.00 versus 7.45 ± 2.01 in the obese group (p < 0.001). Supplementary Table S-3 provides detailed comparisons of the distribution and mean HIS scores between obese and non-obese groups.

**Table 2.** Comparison of HIS Subscale Scores between Obese and Non-Obese Groups.

(N＝2,260)					
Subscale	Obese (Mean ± SD)	Non-obese (Mean ± SD)	Mann-Whitney U	Z	*p*-value
Health Consciousness	6.95 ± 2.22	7.78 ± 2.17	949,400	-10.42	**p<0.001**
Health Motivation	7.27 ± 1.97	7.70 ± 1.99	1,058.390	-6.13	**p<0.001**
Health Value	7.45 ± 2.01	8.15 ± 2.00	970,370	-9.52	**p<0.001**

Mann-Whitney U test was used for comparison between groups

### Univariable linear regression analysis of HIS subscales and BMI

As shown in Table 3, All subscales exhibited significant negative associations with BMI. Among participants with obesity-related diseases, the coefficients for Health Consciousness, Health Motivation, and Health Value were −0.386, −0.436, and −0.345, respectively (p < 0.001). For those without such diseases, the coefficients were −0.268, −0.090, and −0.296, respectively (p < 0.05 to p < 0.001).

**Table 3.** Univariable Linear Regression Analysis of HIS Subscales and BMI.

(N＝2,260)						
HIS subscale	Obesity-related diseases status	B (Coefficient)	95%CI		Standardised Beta	*p*-value
			Lower	Upper		
Health Consciousness	Total	-0.096	-0.120	-0.072	-0.166	<0.001
	Present	-0.386	-0.552	-0.219	-0.205	<0.001
	Absent	-0.268	-0.341	-0.195	-0.167	<0.001
Health Motivation	Total	-0.048	-0.069	-0.026	-0.092	<0.001
	Present	-0.436	-0.620	-0.252	-0.210	<0.001
	Absent	-0.090	-0.173	-0.008	-0.051	<0.05
Health Value	Total	-0.089	-0.111	-0.068	-0.169	<0.001
	Present	-0.345	-0.529	-0.162	-0.168	<0.001
	Absent	0.296	-0.376	-0.215	-0.168	<0.001

### Multivariate logistic regression analysis of HIS subscale scores and obesity

Table 4 presents models without adjustment for obesity-related diseases, while Table 5 shows results adjusted for obesity-related diseases. Supplementary Table S-4 presents results for HIS total score without adjustment for obesity-related diseases.

**Table 4.** Multivariable Logistic Regression Analysis Results of HIS Subscales by Obesity Status (Unadjusted for Obesity-Related Diseases).

(N=2,260)													
Variable	Categorisation criteria	Health Consciousness				Health Motivation				Health Value			
		Odds Ratio	95%CI		*p*-value	Odds Ratio	95%CI		*p*-value	Odds Ratio	95%CI		*p-*value
			Lower	Upper			Lower	Upper			Lower	Upper	
Subscales													
	Continuous variables	0.84	0.81	0.88	**<0.001**	0.91	0.87	0.95	**<0.001**	0.85	0.81	0.89	**<0.001**
Sex													
	Female	Ref.				Ref.				Ref.			
	Male	2.06	1.52	2.79	**<0.001**	2.12	1.57	2.86	**<0.001**	1.99	1.47	2.70	**<0.001**
Age													
	Continuous variables	1.02	1.00	1.03	**<0.05**	1.01	0.99	1.02	0.19	1.02	1.00	1.03	**<0.05**
Occupational categories													
	Less sedentary occupations	Ref.				Ref.				Ref.			
	More sedentary occupations	1.24	1.03	1.49	**<0.05**	1.24	1.03	1.48	**<0.05**	1.25	1.04	1.50	**<0.05**
Job Position Categories													
	General staff	Ref.				Ref.				Ref.			
	Management staff	0.90	0.67	1.21	0.50	0.90	0.68	1.21	0.50	0.91	0.68	1.22	0.53
Presence of co-resident family members													
	Yes	Ref.				Ref.				Ref.			
	No	1.19	0.93	1.54	0.17	1.18	0.92	1.51	0.20	1.19	0.93	1.53	0.17
Marital status													
	Yes	Ref.				Ref.				Ref.			
	No	1.12	0.86	1.45	0.42	1.15	0.88	1.49	0.22	1.13	0.87	1.47	0.35
Daily sleep duration													
	7 hours or more	Ref.				Ref.				Ref.			
	Less than 7 hours	1.33	1.08	1.63	**<0.05**	1.35	1.10	1.66	**<0.001**	1.29	1.05	1.59	**<0.05**
Smoking													
	Non-smoker or quit	Ref.				Ref.				Ref.			
	Smoker	1.10	0.86	1.41	0.45	1.17	0.91	1.49	0.22	1.07	0.84	1.38	0.58
Daily walking time													
	60 minutes or more	Ref.				Ref.				Ref.			
	Less than 60 minutes	0.95	0.69	1.30	0.74	1.02	0.74	1.39	0.92	0.98	0.72	1.35	0.92
Breakfast intake													
	Daily or occasionally	Ref.				Ref.				Ref.			
	None	1.10	0.90	1.34	0.35	1.19	0.98	1.44	0.08	1.17	0.96	1.43	0.11
Snack intake													
	Never or sometimes	Ref.				Ref.				Ref.			
	Every day	0.66	0.51	0.87	**<0.05**	0.68	0.53	0.88	**<0.001**	0.69	0.53	0.90	**<0.001**

Outcome: Obesity status (obese vs. non-obese). HIS Subscales: Health Consciousness, Health Motivation, and Health Value were treated as continuous variables, with the odds ratio representing the change in obesity risk per 1-point increase on a scale of 0-12. Age was treated as a continuous variable, with the odds ratio representing the change in obesity risk per 1-year increase. Ref. denotes the reference group for each categorical variable in the logistic regression analysis.

**Table 5.** Multivariable Logistic Regression Analysis Results of HIS Subscales by Obesity Status (Adjusted for Obesity-Related Diseases).

(N=2,260)													
Variable	Categorisation criteria	Health Consciousness				Health Motivation				Health Value			
		Odds Ratio	95%CI		*p*-value	Odds Ratio	95%CI		*p*-value	Odds Ratio	95%CI		*p*-value
			Lower	Upper			Lower	Upper			Lower	Upper	
Subscales													
	Continuous variables	0.84	0.80	0.88	**<0.001**	0.91	0.87	0.95	**<0.001**	0.85	0.81	0.89	**<0.001**
Sex													
	Female	Ref.				Ref.				Ref.			
	Male	1.84	1.36	2.50	**<0.001**	1.90	1.40	2.57	**<0.001**	1.79	1.32	2.44	**<0.001**
Age													
	Continuous variables	1.00	0.99	1.02	0.64	0.99	0.98	1.01	0.55	1.00	0.99	1.02	0.87
Occupational categories													
	Less Sedentary occupations	Ref.				Ref.				Ref.			
	More Sedentary occupations	1.25	1.03	1.51	**<0.05**	1.25	1.03	1.50	**<0.05**	1.25	1.04	1.51	**<0.05**
Job position categories													
	General staff	Ref.				Ref.				Ref.			
	Management staff	0.93	0.69	1.26	0.64	0.93	0.69	1.25	0.64	0.94	0.69	1.27	0.67
Presence of co-resident family members													
	Yes	Ref.				Ref.				Ref.			
	No	1.15	0.89	1.49	0.30	1.13	0.88	1.46	0.34	1.15	0.89	1.49	0.29
Marital status													
	Yes	Ref.				Ref.				Ref.			
	No	1.07	0.82	1.41	0.62	1.11	0.85	1.45	0.46	1.09	0.83	1.43	0.53
Daily sleep duration													
	7 hours or more	Ref.				Ref.				Ref.			
	Less than 7 hours	1.39	1.12	1.72	**<0.05**	1.40	1.14	1.73	**<0.05**	1.34	1.09	1.66	**<0.05**
Smoking													
	Non-smoker or quit	Ref.				Ref.				Ref.			
	Smoker	1.10	0.86	1.42	0.45	1.19	0.92	1.52	0.19	1.09	0.85	1.41	0.49
Daily walking time													
	60 minutes or more	Ref.				Ref.				Ref.			
	Less than 60 minutes	0.94	0.68	1.29	0.70	1.01	0.73	1.39	0.96	0.98	0.71	1.35	0.89
Breakfast intake													
	Daily or occasionally	Ref.				Ref.				Ref.			
	None	1.09	0.89	1.33	0.43	1.18	0.97	1.44	0.09	1.17	0.96	1.42	0.13
Snack intake													
	Never or sometimes	Ref.				Ref.				Ref.			
	Everyday	0.69	0.52	0.90	**<0.05**	0.71	0.54	0.92	**<0.05**	0.72	0.55	0.94	**<0.05**
Obesity-related diseases													
	No	Ref.				Ref.				Ref.			
	Yes	3.04	2.43	3.79	**<0.001**	2.96	2.38	3.68	**<0.001**	2.94	2.36	3.67	**<0.001**

HIS Subscales: Health Consciousness, Health Motivation, and Health Value were treated as continuous variables, with the odds ratio representing the change in obesity risk per 1-point increase on a scale of 0-12. Age was treated as a continuous variable, with the odds ratio representing the change in obesity risk per 1-year increase. Ref. denotes the reference group for each categorical variable in the logistic regression analysis.

### Obesity-related diseases

The presence of obesity-related diseases was strongly associated with all HIS dimensions. For Health Consciousness, the odds ratio (OR) was 3.04 (2.43-3.79, p < 0.001). Similar results were observed for Health Motivation (OR 2.96, 2.38-3.68, p < 0.001) and Health Value (OR 2.94, 2.36-3.67, p < 0.001).

### HIS subscales

The OR for the HIS subscales was consistent across both models. For Health Consciousness, the OR was 0.84 (unadjusted: 0.81-0.88, adjusted: 0.80-0.88, both p < 0.001). Similarly, Health Motivation had an OR of 0.91 (0.87-0.95, p < 0.001), and Health Value had an OR of 0.85 (0.81-0.89, p < 0.001) in both models.

### Sex

Male participants demonstrated higher odds across all HIS dimensions in the adjusted models. For Health Consciousness, the OR was 1.84 (1.36-2.50, p < 0.001). Similarly, the ORs were 1.90 (1.40-2.57, p < 0.001) for Health Motivation and 1.79 (1.32-2.44, p < 0.001) for Health Value.

### Age

In the unadjusted model, age was significantly associated with Health Consciousness and Health Value, with an OR of 1.02 (1.00-1.03, p < 0.05). However, after adjustment for obesity-related diseases, these associations were no longer significant. No significant association was observed for Health Motivation in either model.

### Occupational categories

Participants in more sedentary occupations had higher odds across all HIS dimensions compared to those in less sedentary occupations. For Health Consciousness, the OR was 1.25 (1.03-1.51, p < 0.05) in the adjusted model. Similar results were observed for Health Motivation (OR 1.25, 1.03-1.50, p < 0.05) and Health Value (OR 1.25, 1.04-1.51, p < 0.05).

### Daily sleep duration

Shorter sleep durations (<7 hours) were significantly associated with higher odds across all HIS dimensions. For Health Consciousness, the OR was 1.39 (1.12-1.72, p < 0.05) in the adjusted model. Similarly, the ORs were 1.40 (1.14-1.73, p < 0.05) for Health Motivation and 1.34 (1.09-1.66, p < 0.05) for Health Value.

### Snack intake

Daily snack intake was associated with lower odds across all HIS dimensions. For Health Consciousness, the OR was 0.69 (0.52-0.90, p < 0.05) in the adjusted model. Similarly, the ORs were 0.71 (0.54-0.92, p < 0.05) for Health Motivation and 0.72 (0.55-0.94, p < 0.05) for Health Value.

Other variables showed no significant associations with any HIS dimension in either model.

Stratified analyses were conducted based on obesity-related disease status, with separate multivariable logistic regression models applied to examine the associations between HIS subscales and obesity in each group.

Supplementary Tables S-5 to S-9 present detailed results, including study participants’ characteristics stratified by obesity-related diseases and multivariable logistic regression analysis results of HIS subscales by obesity status.

## Discussion

### The association between HIS subscales and obesity interventions

In East Asia, including Japan, the average BMI is lower than that in Western countries. However, a BMI exceeding 25 is associated with a 39% increase in mortality risk ^(25)^, emphasizing the need for early-stage intervention. At the pre-obese stage, as defined by the WHO obesity criteria, symptoms are often minimal, making behavioral change challenging when medical treatment is not yet required. To address this issue, this study focused on health interest, a key factor in preventive health behaviors.

The HIS, based on Jayanti and Burn’s Preventive Health Care Behavior Model, comprises subscales that represent conceptual constructs directly associated with health behaviors ^(13)^. To elucidate how different aspects of health interest are associated with obesity and lifestyle behaviors, each subscale was analyzed individually.

Multivariate logistic regression analysis identified significant associations between each HIS subscale and obesity risk. Among these, health consciousness was more strongly associated with reduced obesity risk than health motivation.

Even after adjusting for the presence of obesity-related diseases, the associations between HIS subscales and obesity risk remained significant. This finding suggests that these three concepts are independently associated with obesity risk, regardless of the presence of obesity-related diseases.

These factors have been examined individually for their impact on health behaviors. For instance, previous studies have shown that health consciousness promotes health behaviors and positively influences healthy lifestyles ^(26), (27)^. Although conceptually distinct, a related study found that children of parents with high health awareness tend to have lower BMIs compared to those of parents with low health awareness ^(28)^.

Additionally, health motivation and health value have been reported to promote healthy behaviors ^(13), (29), (30)^.

These findings suggest that future intervention programs should incorporate approaches that consider these characteristics, emphasizing the importance of education and motivational strategies in enhancing health consciousness and fostering health values. These approaches may lead to more effective strategies for obesity prevention.

### Prevention strategies based on obesity-related characteristics

Logistic regression analysis revealed that being male and having a sedentary occupation were significantly associated with an increased risk of obesity. These findings align with previous studies showing that men are at a higher risk of obesity than women ^(31), (32)^. In this study, no significant associations were observed between co-resident family members, marital status, and obesity. Previous research has shown that individuals living alone are more likely to be obese ^(33)^. However, the association between marital status and obesity risk remains controversial, with some research suggesting that single individuals have a lower risk of obesity ^(34)^, whereas other studies indicate that married women are more likely to experience an increase in BMI ^(35)^. Therefore, further investigation is necessary.

Individuals engaged in sedentary work are at an increased risk of obesity ^(17)^. Notably, Japan has the longest sitting time globally ^(36)^, making it crucial to address this issue to prevent obesity and reduce other health risks.

Additionally, lifestyle factors, such as sleeping less than 7 hours, smoking, and skipping breakfast are all associated with an increased risk of obesity ^(37), (38), (39), (40)^. However, daily snacking reduces the risk of obesity ^(41), (42)^, suggesting that moderate snacking may help prevent overeating. These results indicate that improving basic lifestyle behaviors, particularly reducing sedentary behaviors, is crucial for preventing obesity. Effective obesity prevention strategies should be developed to improve these behaviors.

### Weight stigma as a barrier to obesity behavior change

When implementing targeted interventions for obesity prevention, it is essential to address the barriers faced by individuals living with obesity. Weight stigma, defined as pervasive misconceptions and stereotypes about higher body weight ^(43)^, has been associated with obesity and increased diabetes risk ^(44)^, as well as psychological factors such as stress and anxiety ^(44), (45)^. Furthermore, weight stigma contributes to disordered eating behaviors, including binge eating ^(46), (47)^, and reduces motivation for physical activity ^(46)^ and actual participation in physical activity ^(48), (49), (50)^.

Individuals living with obesity may exhibit high levels of health interest but remain adversely affected by weight stigma and other barriers. Even after adjusting for BMI, weight stigma has been shown to disrupt lifestyle behaviors, such as promoting disordered eating ^(48)^. These complexities highlight the importance of carefully considering such factors when developing interventions to address obesity.

### Health interest and BMI in individuals with obesity-related diseases

Table 3 shows significant negative associations between the three HIS subscales and BMI, especially in participants with obesity-related diseases. These subscales are more effective in reducing BMI in this group, with weaker associations in those without such diseases. The role of health consciousness in promoting dietary compliance, as noted by Kapur et al. ^(51)^ and Kato et al. ^(52)^, underscores the importance of enhancing health interest, particularly in individuals with obesity-related diseases. Additionally, Clark et al. ^(53)^ highlighted the significance of diabetes empowerment and motivation, while Nugent et al. ^(54)^ emphasized health value and control over health in self-management, reinforcing the need for targeted interventions.

### Limitations of the study

This cross-sectional study has limitations, including the inability to infer causality and missing data. Future research should address these issues. Despite these limitations, the findings provide valuable insights into health interests and obesity risk, supporting targeted prevention strategies.

### Conclusions

This study found significant associations between the three HIS dimensions and obesity risk. Among these, health consciousness and health value were particularly strongly associated with reduced risk. Male sex and sedentary occupations were associated with higher risk. These associations remained significant after adjusting for obesity-related diseases, especially in participants with such diseases. This highlights the need for targeted interventions.

## Article Information

### Conflicts of Interest

None

### Sources of Funding

JST SPRING (grant number JPMJSP2110).

### Acknowledgement

We are deeply grateful to Mr. Yasuhiro Azuma (Senior Advisor), Mr. Hirohisa Kato (Manager), Ms. Mutsumi Okada (Director of the Health Support Office), and Ms. Kumiko Tsutsui (Manager) at Fujitsu Limited for their cooperation in data collection and provision. We also extend our sincere appreciation to Associate Professor Misa Shiomi at Kyoto University for her dedicated efforts and invaluable contribution to this investigation.

### Author Contributions

Yumiko Iwase and Rikuya Hosokawa contributed to the conception, design, analysis, and preparation of the manuscript. Data collection and aggregation were conducted by the team at Fujitsu Limited. Yumiko Iwase performed the statistical analysis. All authors read and approved the final manuscript.

### Approval by Institutional Review Board (IRB)

All procedures were performed in accordance with Kyoto University Hospital’s ethical standards and the Declaration of Helsinki. This study was approved by the Institutional Review Board of Kyoto University Hospital (approval code: R4128).

### Informed Consent

The research information sheet was used to explain the study to all participants before they answered the questionnaire and signed a consent form.

## Supplement

Supplementary Tables The online version contains supplementary material, including Tables S1-S9, which provide detailed analyses as follows:
・Table S-1: Reliability analysis results of the HIS.
・Table S-2: Comparison of HIS scores by participant characteristics.
・Table S-3: Distribution and mean HIS scores among obese and non-obese groups.
・Table S-4: Multivariable logistic regression analysis results of HIS total score by obesity status (Unadjusted for obesity-related diseases)
・Table S-5: Characteristics of the study participants: No obesity-related diseases.
・Table S-6: Characteristics of the study participants: With obesity-related diseases.
・Table S-7: Comparison of HIS total and subscale scores by the presence of obesity-related diseases
・Table S-8: Multivariable logistic Regression Analysis Results of HIS Subscales by obesity status: No obesity-related diseases.
・Table S-9: Multivariable logistic regression analysis results of HIS subscales by obesity status: With obesity-related diseases.
These materials are available in PDF format for further reference.
