# Supplementary material for: Associations between Health Interest Scale Dimensions and Obesity Risk: A Cross-sectional Study among Japanese Employees
Source: JMA J. 2025 Mar 28;8(2):453–64. doi: 10.31662/jmaj.2024-0388 (PMC12095852; doi:10.31662/jmaj.2024-0388)
Supplement: Supplementary Tables — The online version contains supplementary material, including Tables S1-S9, which provide detailed analyses as follows: ・Table S-1: Reliability analysis results of the HIS. ・Table S-2: Comparison of HIS scores by participant characteristics. ・Table S-3: Distribution and mean HIS scores among obese and non-obese groups. ・Table S-4: Multivariable logistic regression analysis results of HIS total score by obesity status (Unadjusted for obesity-related diseases) ・Table S-5: Characteristics of the study participants: No obesity-related diseases. ・Table S-6: Characteristics of the study participants: With obesity-related diseases. ・Table S-7: Comparison of HIS total and subscale scores by the presence of obesity-related diseases ・Table S-8: Multivariable logistic Regression Analysis Results of HIS Subscales by obesity status: No obesity-related diseases. ・Table S-9: Multivariable logistic regression analysis results of HIS subscales by obesity status: With obesity-related diseases. These materials are available in PDF format for further reference. [file 2433-3298-8-2-0453-s001.pdf]

Table.S-1: Reliability analysis results of the HIS (N=2,260)

Subscale	Item	Mean	SD	Scale mean if item deleted	Scale variance if item deleted	Corrected item-total correlation	Cronbach Alpha if item deleted	Cronbach Alpha
Health Consciousness	1. I am very self-conscious about my health.	1.74	0.688	21.30	20.826	0.643	0.843	0.85
	2. I am interested in information about my health.	2.17	0.611	20.86	21.557	0.599	0.846	
	3. I pay attention to changes in my health condition.	2.03	0.633	21.00	21.316	0.619	0.845	
	4. I am more health conscious than people around me.	1.58	0.713	21.46	20.695	0.637	0.843	
Health Motivation	5. I am willing to spend some extra money for my health.	2.09	0.564	20.95	22.030	0.564	0.849	0.78
	6. I do everything I can to stay healthy.	2.01	0.608	21.03	21.909	0.537	0.850	
	7. We should spend some extra time for health.	2.07	0.678	20.96	21.144	0.598	0.846	
	8. I want to put health first in my living	1.43	0.705	21.61	21.282	0.547	0.850	
Health Value	9. Work and income are more important than health.	1.68	0.666	21.36	22.326	0.407	0.859	0.69
	10. I worry about my health only when I get sick.	1.95	0.661	21.09	22.510	0.381	0.860	
	11. Hobbies and leisure activities are more important than health.	2.29	0.636	20.74	22.109	0.472	0.854	
	12. Rather than prevent illness, it is best just to cure it when I get sick.	2.00	0.813	21.04	20.960	0.499	0.854	
HIS		23.04	5.03					0.86

The item translations in this table are based on Wakabayashi (2023).

TableS-2. Comparison of HIS scores by participant characteristics (N=2,260)

Variable	N	(%)	Mean	SD	95%CI		Comparison with 39 years and under		Comparison with 40 to 49 years	
					Lower	Upper	Mean Difference	*p* -value	Mean Difference	*p*- value
Age (years)										
≤39	115	5.1	21.7	4.79	20.8	22.6	-	-	-0.75	0.29
40–49	809	35.8	22.4	4.95	22.1	22.8	0.75	0.29	-	-
≥50	1336	59.1	23.5	5.05	23.2	23.7	1.78	**<0.05**	1.03	**<0.001**
Total	2260	100	23.0	5.03	22.8	23.2	-	-	-	-

Variable	N	(%)	Mean	SD	t value	*p*- value
Sex						
Female	332	14.7	24.1	5.00	4.39	**<0.001**
Male	1928	85.3	22.8	5.01		
Occupational categories						
Less sedentary occupations	980	43.4	23.2	5.11	1.60	0.109
More sedentary occupations	1280	56.6	22.9	4.97		
Job position categories						
General staff	2015	89.2	23.0	5.07	-0.53	0.595
Management staff	245	10.8	23.2	4.73		
Marital status						
Yes	1709	75.6	23.2	4.99	2.75	**<0.05**
No	551	24.4	23.2	5.15		
Presence of co-resident family members						
Yes	1657	75.3	23.2	4.94	3.05	**<0.05**
No	603	26.7	22.5	5.23		
Smoking						
Non-smoker or quit	1907	84.4	23.4	4.91	9.70	**<0.001**
Smoker	353	15.6	20.7	5.03		
Daily walking time						
≥60 min	201	8.9	24.0	5.74	2.46	**<0.05**
<60 min	2059	91.1	22.9	4.95		
Daily sleep duration						
≥7 h	645	28.5	23.4	5.05	2.50	**<0.05**
<7 h	1615	71.5	22.8	5.02		
Breakfast intake						
Daily or occasionally	1469	65.0	23.7	4.97	8.45	**<0.001**
None	791	35.0	21.8	4.93		
Snack intake						
Never or sometimes	1877	83.1	23.0□	5.01	0.13	0.895
Every day	383	16.9	23.0	5.15		
Obesity status						
Non-obesity	1498	66.3	23.7	4.97	-1.96	**<0.001**
Obesity	762	33.7	21.7	4.90		

Sedentary occupations: SE/CE, Clerical worker

Management staff: Director, Executive director or above

Table.S-3: Distribution and mean HIS scores among obese and non-obese groups (N=2,260)

	Total	Obesity	Non Obesity	*p* -value
The mean score of HIS (SD)	23.01(5.03)	21.71(4.99)	24.00(4.97)	<0.001†
Health indifference (0-18), N (%)	556	258(46.4%)	298(53.6%)	
Low health interest (19-21), N (%)	498	194 (39.0%)	304(61.0%)	
Middle health interest (22-23), N (%)	364	108(29.7%)	256(70.3%)	<0.001‡
Middle-high health interest (24-27), N (%)	422	101(23.9%)	321(76.1%)	
High health interests (28-36), N (%)	420	101(24.0%)	319(76.0%)	
Total	2260	762(33.7%)	1498(66.3%)	

† : t-test

‡ : χ^2test

Table.S-4: Multivariable logistic regression analysis results of HIS total score by obesity status (Unadjusted for obesity-related diseases) (N = 2260)

Variable	Categorization criteria	Odds Ratio	95%CI Lower	95%CI Upper	*p* -value
Health interest score by HIS(Continuous variable)					
		0.93	0.91	0.95	**<0.001**
Age (years) (Continuous variable)					
		1.02	1.00	1.03	**<0.05**
Sex					
	Female		Ref.		
	Male	2.00	1.48	2.71	**<0.001**
Occupational categories					
	Less sedentary occupations		Ref.		
	More sedentary occupations	1.23	1.02	1.48	**<0.05**
Job position categories					
	General staff		Ref.		
	Management staff	0.91	0.68	1.23	0.55
Presence of co-resident family members					
	Yes		Ref.		
	No	1.17	0.91	1.51	0.22
Marital status					
	Yes		Ref.		
	No	1.13	0.86	1.47	0.38
Daily sleep duration					
	7 hours or more		Ref.		
	Less than 7 hours	1.31	1.07	1.62	**<0.05**
Smoking					
	Non-smoker or quit		Ref.		
	Smoker	1.04	0.81	1.34	0.76
Daily walking time	60 minutes or more		Ref.		
	Less than 60 minutes	0.97	0.70	1.33	0.84
Breakfast intake	Daily or occasionally		Ref.		
	None	1.12	0.92	1.36	0.26
Snack intake	Never or sometimes		Ref.		
	Every day	0.67	0.51	0.87	**<0.05**

Dependent variable:Obesity

Sedentary occupations: SE/CE, Clerical worker

Management staff: Director, Executive director or above

Ref. denotes the reference group for each categorical variable in the logistic regression analysis.

TableS-5. Characteristics of the study participants: **No obesity related diseases** (n＝1,787)

Categorical variable	Categorisation criteria	Total n (%)	Obesity n (%)	Non-obesity n (%)	Effect size (Cramer'sV)	Chi-square test (*p*-value)
Sex, n (%)						
	Male	1,489(83.3%)	451(89.7%)	1038(80.8%)	0.106	**<0.001**
	Female	298(16.7%)	52(10.3%)	246(19.2%)		
Occupational categories, n (%)						
	Less sedentary occupations	774(43.3%)	203(40.4%)	571(44.5%)	0.037	0.115
	More sedentary occupations	1,013(56.7%)	300(59.6%)	713(55.5%)		
Job/Position categories, n (%)						
	General staff	1,591(89.0%)	454(90.3%)	1,137(88.6%)	0.025	0.299
	Management staff	196(11.0%)	49(9.7%)	147(11.4%)		
Presence of co-resident family members, n (%)						
	Yes	1,339(73.9%)	360(71.6%)	979(76.2%)	0.049	**<0.05**
	No	448(25.1%)	143(28.4%)	305(23.8%)		
Marital status, n (%)						
	Yes	1,371(76.7%)	373(74.2%)	998(77.7%)	0.038	0.108
	No	416(23.3%)	130(25.8%)	286(22.3%)		
Age categories, n (%), years						
	≤39	106(5.9%)	29(5. 7 %)	77(6.0%)	0.025	0.566
	40–49	697(39.0%)	187(37.2%)	510(39.7%)		
	≥50	984(55.1%)	287(57.1%)	697(54.3%)		
WHO Obesity classification	BMI classification	n	(%)			

Underweight (<18.5)	92	(5.1%)
Normal (18.5–24.9)	1192	(66.7%)
Pre-obese (25.0–29.9)	421	(23.6%)
Obese Class I (30.0–34.9)	70	(3.9%)
Obese Class II (35.0–39.9)	9	(0.5%)
Obese Class III (≥40.0)	3	(0.2%)

Continuous variable	Mean±SD
Age, years	49.99±6.84
BMI, kg/m^2	23.46±3.53
HIS Score	23.05±5.00
Health Consciousness	7.51±2.21
Health Motivation	7.61±1.98
Health Value	7.93±2.01

Occupational categories: "More sedentary occupations" include System engineer/Customer engineer (SE/CE) and Clerical workers. "Less sedentary occupations" include all other roles.

Job position categories: "Management staff" includes Director, Executive director, or higher positions. "General staff" includes all other positions.

HIS score: The HIS is a validated 12-item scale that assesses health interest across three subscales: health consciousness, motivation, and value. Each item is rated on a 4-point Likert scale (0–3), with reverse scoring for items 9–12. Total scores range from 0 to 36

TableS-6. Characteristics of the study participants: **With obesity related diseases** (n=473)

Categorical variable	Categorisation criteria	Total n (%)	Obesity n (%)	Non-obesity n (%)	Effect size (Cramer'sV)	Chi-square test (*p*-value)
Sex, n (%)						
	Male	439(92.8%)	245(94.6%)	194(90.7%)	0.076	**<0.05**
	Female	34(7,2%)	14(5.4%)	20(9.3%)		
Occupational categories, n (%)						
	Less sedentary occupations	206(43.9%)	102(39.4%)	104(48.6%)	0.093	**<0.05**
	More sedentary occupations	267(56.1%)	157(60.6%)	110(51.4%)		
Job/position categories, n (%)						
	General staff	424(89.6%)	228(88.0%)	196(91.6%)	0.058	0.206
	Management staff	49(10.4%)	31(12.0%)	18(8.4%)		
Presence of co-resident family members, n (%)						
	Yes	318(67.2%)	166(64.1%)	152(71.0%)	0.074	0.110
	No	155(32.8%)	93(35.9%)	62(29.0%)		
Marital status, n (%)						
	Yes	338(71.5%)	180(69.5%)	158(73.8%)	0.048	0.299
	No	135(28.5%)	79(30.5%)	56(26.2%)		
Age categories, n (%), years						
	≤39	9(1.9%)	7(2.7%)	2(0.9%)	0.065	0.369
	40–49	112(23.7%)	60(23.2%)	52(24.3%)		
	≥50	352(74.4%)	192(74.1%)	160(74.8%)		
WHO Obesity classification	BMI classification	n	(%)			
	Underweight (<18.5)	6	(1.3%)			

	Normal (18.5–24.9)	208	(44.0%)
	Pre-obese (25.0–29.9)	190	(40.2%)
	Obese Class I (30.0–34.9)	52	(11.0%)
	Obese Class II (35.0–39.9)	15	(3.2%)
	Obese Class III (≥40.0)	2	(0.4%)
Continuous Variable	Mean±SD		
Age, years	53.28± 6.07		
BMI, kg/m^2	25.96± 4.19		
HIS Score	22.87±5.15		
Health Consciousness	7.59±2.23		
Health Motivation	7.51±2.02		
Health Value	7.79±2.04		

Occupational categories: "More sedentary occupations" include System engineer/Customer engineer (SE/CE) and Clerical workers. "Less sedentary occupations" include all other roles.

Job position categories: "Management staff" includes Director, Executive director, or higher positions. "General staff" includes all other positions.

HIS score: The HIS is a validated 12-item scale that evaluates health interest across three subscales: health consciousness, health motivation, and health value. Each item is rated on a 4-point Likert scale (0–3), with reverse scoring applied to items 9–12. Total scores range from 0 to 36.

TableS-7. Comparison of HIS total and subscale scores by the presence of obesity-related diseases

Subscale	No obesity-related diseases (Mean ± SD)	With obesity-related diseases (Mean ± SD)	Mann-Whitney U	Z	*p*-value
HIS total	23.05 ± 5.00	22.87 ± 5.15	416,138	-0.52	0.607
Health Consciousness	7.51 ± 2.21	7.59 ± 2.23	416,179	-0.52	0.605
Health Motivation	7.61 ± 1.98	7.51± 1.99	410,786	-0.95	0.341
Health Value	7.93 ± 2.01	7.79 ± 2.04	404,541	-1.36	0.173

Mann–Whitney U test was used for comparison between groups

TableS-8. Multivariable logistic regression analysis results of HIS subscales by obesity status **(No Obesity-related Diseases)** (n＝1,787)

Variable	Categorisation criteria	Health Consciousness				Health Motivation				Health Value			
		Odds Ratio	95%CI		*p*-value	Odds Ratio	95%CI		*p*-value	Odds Ratio	95%CI		*p*-value
			Lower	Upper			Lower	Upper			Lower	Upper	
Subscales													
	Continuous variables	0.850	0.807	0.895	<0.001	0.957	0.907	1.010	0.112	0.858	0.812	0.907	**<0.001**
Sex													
	Female		Ref.				Ref.				Ref		
	Male	1.877	1.338	2.635	**<0.001**	1.954	1.396	2.734	**<0.001**	1.812	1.291	2.543	**<0.05**
Age													
	Continuous variables	1.005	0.989	1.022	0.542	0.998	0.982	1.014	0.799	1.003	0.987	1.019	0.727
Occupational categories													
	Less sedentary occupations		Ref.				Ref.				Ref.		
	More sedentary occupations	1.183	0.951	1.472	0.131	1.175	0.946	1.458	0.145	1.200	0.965	1.492	0.101
Job position categories													
	General staff		Ref.				Ref.				Ref.		
	Management staff	0.767	0.538	1.093	0.142	0.768	0.541	1.092	0.141	0.775	0.544	1.103	0.157
Presence of co-resident family members													
	Yes		Ref.				Ref.				Ref.		
	No	1.089	0.802	1.480	0.585	1.100	0.813	1.487	0.537	1.095	0.808	1.485	0.557
Marital status													
	Yes		Ref,				Ref.				Ref.		
	No	0.474	1.124	0.807	0.895	1.154	0.842	1.581	0.373	1.149	0.836	1.578	0.392
Daily sleep duration													
	7 hours or more		Ref.				Ref.				Ref.		

	Less than 7 hours	1.412	1.102	1.808	**<0.05**	1.449	1.134	1.852	**<0.05**	1.383	1.080	1.771	**<0.05**
Smoking													
	Non-smoker or quit			Ref.				Ref.				Ref.	
	Smoker	1.197	0.896	1.600	0.224	1.311	0.894	1.746	0.064	1.163	0.868	1.557	0.312
Daily walking time													
	60 Minutes or more			Ref.				Ref.				Ref.	
	Less than 60 minutes	1.079	0.733	1.589	0.699	1.159	0.792	1.695	0.448	1.120	0.763	1.645	0.563
Breakfast intake													
	Daily or occasionally			Ref.				Ref.				Ref.	
	None	0.930	0.737	1.174	0.543	1.010	0.804	1.270	0.931	0.978	0.777	1.232	0.851
Snack intake													
	Never or sometimes			Ref.				Ref.				Ref.	
	Every day	0.634	0.467	0.862	**<0.05**	0.650	0.480	0.880	**<0.05**	0.650	0.480	0.882	**<0.05**

Outcome: Obesity status (obese vs. non-obese).

HIS Subscales: Health Consciousness, Health Motivation, and Health Value were treated as continuous variables, with the odds ratio representing the change in obesity risk per 1-point increase on a scale of 0–12.

Age was treated as a continuous variable, with the odds ratio representing the change in obesity risk per 1-year increase.

Ref. denotes the reference group for each categorical variable in the logistic regression analysis.

TableS-9. Multivariable logistic regression analysis results of HIS subscales by obesity status (**With obesity-related diseases**) (n=473)

Variable	Categorisation criteria	Health Consciousness				Health Motivation				Health Value			
		Odds Ratio	95%CI Lower	95%CI Upper	*p*-value	Odds Ratio	95%CI Lower	95%CI Upper	*p*-value	Odds Ratio	95%CI Lower	95%CI Upper	*p*-value
Subscales													
	Continuous variables	0.797	0.723	0.877	**<0.001**	0.763	0.687	0.848	**<0.001**	0.829	0.750	0.917	**<0.001**
Sex													
	Female		Ref.				Ref.				Ref		
	Male	1.492	0.692	3.217	0.308	1.567	0.714	3.438	0.263	1.544	0.715	3.335	0.269
Age													
	Continuous variables	0.993	0.961	1.026	0.666	0.979	0.947	1.011	0.195	0.992	0.960	1.024	0.610
Occupational categories													
	Less sedentary occupations		Ref.				Ref.				Ref.		
	More sedentary occupations	1.471	0.994	2.178	0.054	1.535	1.035	2.277	**<0.05**	1.409	0.953	2.085	0.086
Job position categories													
	General staff		Ref.				Ref.				Ref.		
	Management staff	1.819	0.942	3.514	0.075	1.925	0.992	3.735	0.053	1.785	0.927	3.438	0.083
Presence of co-resident family members													
	Yes		Ref.				Ref.				Ref.		
	No	1.362	0.817	2.271	0.236	1.289	0.772	2.152	0.331	1.304	0.789	2.157	0.300
Marital status													
	Yes		Ref,				Ref.				Ref.		
	No	0.921	0.543	1.563	0.761	0.907	0.533	1.545	0.720	0.936	0.556	1.576	0.803
Daily sleep duration													
	7 hours or more		Ref.				Ref.				Ref.		

	Less than 7 hours	1.387	0.900	2.137	0.138	1.535	1.035	2.277	**<0.05**	1.270	0.828	1.946	0.273
Smoking													
	Non-smoker or quit			Ref.				Ref.				Ref.	
	Smoker	0.874	0.513	1.488	0.620	0.834	0.488	1.428	0.509	0.929	0.548	1.576	0.786
Daily walking time													
	60 minutes or more			Ref.				Ref.				Ref.	
	Less than 60 minutes	0.628	0.325	1.214	0.167	0.635	0.328	1.231	0.179	0.682	0.357	1.300	0.244
Breakfast intake													
	Daily or occasionally			Ref.				Ref.				Ref.	
	None	1.787	1.165	2.741	**<0.05**	1.935	1.263	2.974	<0.05	2.078	1.366	3.162	**<0.05**
Snack intake													
	Never or sometimes			Ref.				Ref.				Ref.	
	Every day	0.899	0.493	1.639	0.727	0.928	0.508	1.696	0.808	1.045	0.577	1.892	0.884

Outcome: Obesity status (obese vs. non-obese).

HIS Subscales: Health Consciousness, Health Motivation, and Health Value were treated as continuous variables, with the odds ratio representing the change in obesity risk per 1-point increase on a scale of 0–12.

Age was treated as a continuous variable, with the odds ratio representing the change in obesity risk per 1-year increase.

Ref. denotes the reference group for each categorical variable in the logistic regression analysis.
